# 化疗相关性再生障碍性贫血患者的体细胞变异及免疫特征：与未发生再障的肿瘤及原发性再障患者比较

**DOI:** 10.3760/cma.j.cn121090-20251119-00539

**Published:** 2026-05

**Authors:** 一鸣 张, 永鑫 周, 起林 庄, 冰 韩

**Affiliations:** 1 中国医学科学院北京协和医院血液科，北京 100730 Department of Hematology, Peking Union Medical College Hospital, Beijing 100730, China; 2 中国医学科学院北京协和医学院，北京 100730 Chinese Academy of Medical Sciences & Peking Union Medical College, Beijing 100730, China

## Abstract

本研究旨在描述化疗相关性再生障碍性贫血（CAA）患者的体细胞变异候选基因谱，并与未发生AA（non-AA）的肿瘤患者及原发性AA（PAA）患者进行比较。研究纳入2019年9月至2023年5月在北京协和医院确诊的24例CAA患者（男女比3∶5，中位年龄60岁），采集外周血进行全外显子测序，将结果与non-AA肿瘤患者及PAA患者的公开数据进行对比分析。共检出37 111个变异，涉及9 958个基因，KEGG富集分析显示这些基因主要集中于JAK-STAT信号通路、钙离子信号通路等（均*P*<0.01）。在HLA基因方面，CAA患者的HLA-DRB1变异频率高于non-AA肿瘤患者（FDR＝0.029），而HLA-A（FDR＝0.082）和HLA-C（FDR＝0.058）变异频率则低于PAA患者。在髓系疾病相关基因方面，与non-AA肿瘤患者相比，CAA患者中BRCA2（FDR＝0.032）、ASXL1（FDR＝0.047）等198个基因的变异频率更高，SAA2（FDR＝0.049）、TP53（FDR＝0.045）、PIK3CA（FDR＝0.049）等基因的变异频率更低；与PAA患者相比，CAA患者中BRCA2（FDR＝0.068）、ATRX（FDR＝0.072）等213个基因变异频率更高，ASXL1（FDR＝0.045）、DNMT3A（FDR＝0.078）等14个基因变异频率更低。综上，CAA患者的体细胞变异谱与non-AA肿瘤患者及PAA患者存在显著差异：其免疫异常程度高于non-AA肿瘤患者但轻于PAA患者，髓系演变倾向较non-AA肿瘤患者更高，但转化机制较PAA患者更复杂，受原发肿瘤特性及髓系基因变异等多重因素影响。

## Abstract (en)

This study aimed to characterize the somatic variant candidate gene profile of patients with chemotherapy-associated aplastic anemia（CAA）and compare it with that of patients with cancer without aplastic anemia（non-AA）and primary aplastic anemia（PAA）. This study included 24 patients with CAA diagnosed at Peking Union Medical College Hospital from September 2019 to May 2023（male-to-female ratio of 3∶5; median age, 60 years）. Peripheral blood samples were collected for whole-exome sequencing, and the results were compared with publicly available data of patients with non-AA and PAA. A total of 37 111 variants across 9 958 genes were detected. KEGG enrichment analysis revealed that these genes were mainly concentrated in the JAK-STAT and calcium signaling pathways（all *P*<0.01）. Regarding human leukocyte antigen（HLA）genes, the mutation frequency of HLA-DRB1 was higher in patients with CAA than in those with non-AA cancer［false discovery rate（FDR）＝0.029］, whereas the mutation frequencies of HLA-A（FDR＝0.082）and HLA-C（FDR＝0.058）were lower than in those with PAA. For myeloid disease-related genes, compared with patients with non-AA cancer, those with CAA had higher mutation frequencies in 198 genes, including BRCA2（FDR＝0.032）and ASXL1（FDR＝0.047）, and lower frequencies in SAA2（FDR＝0.049）, TP53（FDR＝0.045）, and PIK3CA（FDR＝0.049）. Compared with patients with PAA, those with CAA had higher mutation frequencies in 213 genes, including BRCA2（FDR＝0.068）and ATRX（FDR＝0.072）, and lower frequencies in 14 genes, including ASXL1（FDR＝0.045）and DNMT3A（FDR＝0.078）. In conclusion, the somatic variant profile of CAA significantly differs from that of non AA cancer and PAA: its degree of immune abnormality is higher than that in non-AA cancer but milder than that in PAA; it shows a higher potential for myeloid evolution than non-AA cancer, but its transformation mechanism is more complex than that of PAA, being influenced by multiple factors including primary tumor characteristics and myeloid gene variants.

化疗是多种实体瘤与血液肿瘤的基础治疗之一。然而，化疗药物可通过多种机制导致血细胞减少。多数患者的化疗诱导性骨髓抑制在停药后可逐渐恢复，但仍有部分患者在化疗结束3～6个月后出现不可逆的骨髓功能衰竭[1]，其中一部分最终被诊断为化疗相关性再生障碍性贫血（CAA）。既往研究显示，与单用艾曲泊帕相比，联用环孢素A（CsA）等免疫抑制剂能够显著改善疗效[2]–[3]，提示CAA的发病机制在一定程度上与原发性再生障碍性贫血（PAA）相似。

然而，CAA的具体分子病例机制，以及CAA与未发生AA（non-AA）的肿瘤和PAA之间的分子学特征差异仍不明确。本研究对24例CAA患者进行全外显子测序，重点分析HLA等位基因和与髓系疾病相关基因，并将结果与non-AA肿瘤患者及PAA患者的公开数据进行比较，旨在进一步阐明CAA骨髓衰竭的分子学基础，并为临床管理提供参考。

## 病例与方法

一、病例资料

纳入2019年9月至2023年5月在北京协和医院确诊的CAA患者。入组标准：（1）恶性肿瘤病理学结果经复核确认；（2）化疗前血常规正常，无AA病史；（3）化疗后出现显著血细胞减少，且停药≥6个月未恢复。

收集患者确诊CAA后治疗前的外周血标本；同时收集人口学资料、病史（诊治时间、临床表现、肿瘤类型）、体格检查、既往史、家族史及实验室指标。记录肿瘤治疗经过、CAA发生时间、治疗及转归，疗效评价参照《再生障碍性贫血诊断与治疗中国指南（2022年版）》[4]。本研究经北京协和医院伦理委员会批准（编号：I-23PJ105）。所有患者在入组前均签署知情同意书。

二、基因测序与变异筛选

采用Agilent SureSelect Human All Exon v6试剂盒捕获外周血DNA全外显子区域，经Illumina HiSeq X10平台测序，平均深度200×。基因注释使用ANNOVAR。为聚焦潜在致病变异，剔除最小等位基因频率（MAF）>0.05的变异（参考1000g2015aug_all、gnomad211_genome数据库），随后基于clinvar_20250630数据库的致病性注释，对变异进行致病性分类。后续分析仅保留ACMG 1类和2类变异。

三、关键基因与富集分析

HLA基因共16个，包括经典的Ⅰ类和Ⅱ类基因。髓系疾病相关基因列表来源于DisGeNET平台（筛选标准：基因-疾病关联评分≥0.1）。利用Kyoto Encyclopedia of Genes and Genomes（KEGG）数据库进行通路富集分析，聚焦于变异等位基因频率（VAF）>0.1的变异（即克隆占比>10％，以排除由测序误差或极低丰度亚克隆引入的假阳性信号），借助Metascape平台及Cytoscape进行可视化。HLA分型采用HLAscan（v2.1.2），分辨率达4-digit。

四、对照组数据来源

non-AA肿瘤患者数据来自ICGC数据库（项目编号PTC-SA、LUCA-KR、APGI-AU、PACA-CA），共纳入9种肿瘤类型患者的外周血全外显子组数据：乳腺癌1 182例、肺癌1 140例、胃癌1 114例、结直肠癌828例、子宫内膜癌530例、胰腺癌496例、卵巢癌411例、食管癌315例、宫颈癌289例，共6 305例。PAA数据来自EGA数据库（EGAS00001001153），共186例。

五、统计学处理

使用R 4.1.1进行数据分析。基因变异频率（MF）为群体水平统计指标。两组间比较采用Fisher精确检验，并采用Benjamini-Hochberg法控制假发现率（FDR），显著性阈值设为FDR<0.1。三组间比较中，连续变量（如平均变异数量）采用Kruskal-Wallis *H*检验，分类变量（如携带变异比例）采用Fisher精确检验。双侧*P*<0.05为差异有统计学意义。

## 结果

一、病例特征

共纳入CAA患者24例，其中男9例，女15例，中位年龄60（32～79）岁。肿瘤类型包括：肺癌4例、卵巢癌3例、结肠癌3例、乳腺癌3例、直肠癌2例、宫颈癌2例，以及胰腺癌、胃癌、食管癌、壶腹癌、腹膜癌、子宫内膜癌、滋养层肿瘤各1例。中位化疗4（1～9）个疗程，CAA的诊断在最后一次化疗结束后6～10个月确立。所有患者确诊后均接受过包括雄激素、重组人促红细胞生成素（rhEPO）、重组促血小板生成素（rhTPO）、血小板生成素受体激动剂（TPO-RA）以及IL-11等治疗。部分患者还接受了免疫抑制治疗（IST），包括CsA或他克莫司。全部患者6个月总反应率（ORR）为83.3％。详细肿瘤类型、化疗方案及基线特征见附表1（**扫描本文首页二维码查阅**）。由于样本量有限且肿瘤类型分散，未进行化疗方案或肿瘤危险分层的系统性分组分析。

二、CAA患者基因富集分析

共检出37 111个SNP和INDEL致病/可能致病变异，涉及9 958个基因（non-AA肿瘤组检出29 894个变异，涉及8 773个基因，PAA组检出34 964个变异，涉及9 267个基因），其中1 047个基因的VAF>0.1。KEGG富集分析提示在细胞外基质-受体相互作用、JAK-STAT信号通路、钙离子信号通路等显著富集（图1）。

**图1:**
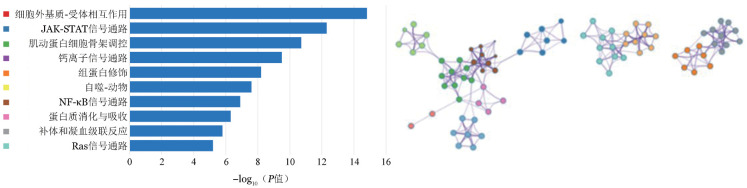
化疗相关性再生障碍性贫血（CAA）患者变异基因的KEGG富集分析（左侧为KEGG富集条形图，右侧为富集网络，网络图中相同颜色的节点代表功能相关性高的通路）

三、CAA患者与对照组HLA基因MF的比较

在16种HLA基因中，与non-AA肿瘤患者相比，CAA患者中HLA-DRB1（FDR＝0.029）基因MF更高（图2A）。与PAA患者相比，CAA患者中HLA-A（FDR＝0.082）、HLA-C（FDR＝0.058）基因MF显著更低（图2B），其余基因（包括HLA-DRB1）差异无统计学意义。

**图2:**
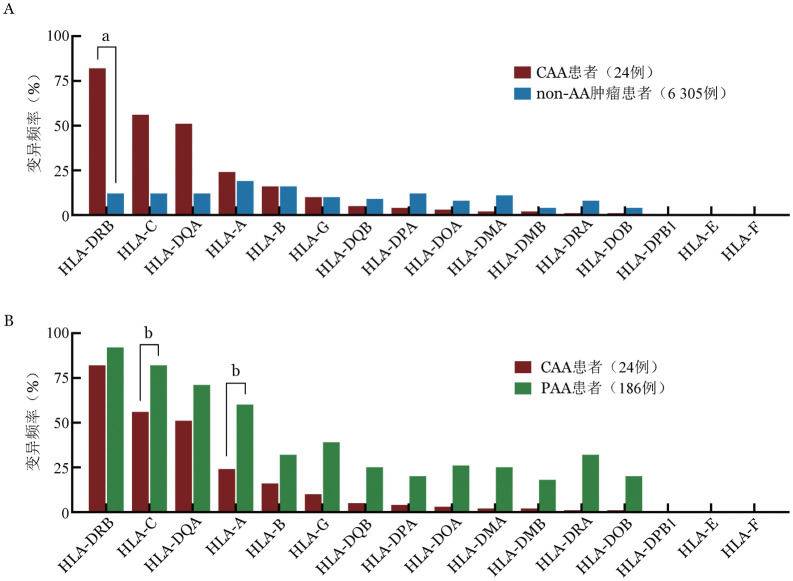
CAA患者与non-AA肿瘤患者（A）及PAA患者（B）HLA基因变异频率比较 **注** CAA患者：化疗相关性再生障碍性贫血患者；non-AA肿瘤患者：未发生再生障碍性贫血的肿瘤患者；PAA患者：原发性再生障碍性贫血患者

四、CAA患者与对照组髓系疾病相关基因MF的比较

在数据库筛选出的1 326个髓系疾病相关基因中，共708个基因检出变异。在携带至少一个髓系相关变异的患者比例方面，CAA组（58.7％）、non-AA肿瘤组（56.1％）与PAA组（56.8％）三组间差异无统计学意义（*P*＝1.000）。在平均变异数量上，CAA组为（1.8 ± 1.3）个，non-AA肿瘤组为（1.7 ± 1.2）个，PAA组为（2.0 ± 1.5）个，三组间差异虽有统计学意义（*H*＝37.56，*P*<0.001），但效应量微弱、临床意义有限。

与non-AA肿瘤患者比较，CAA患者中BRCA2（FDR＝0.032）、ASXL1（FDR＝0.047）、KAT6A（FDR＝0.087）等198个髓系疾病相关变异基因的MF更高，SAA2（FDR＝0.049）、TP53（FDR＝0.045）和PIK3CA（FDR＝0.049）的MF更低，其余基因差异无统计学意义；与PAA患者比较，CAA患者中BRCA2（FDR＝0.068）、RUNX1（FDR＝0.080）、ATRX（FDR＝0.072）等213个髓系疾病相关变异基因MF更高，ASXL1（FDR＝0.045）、DNMT3A（FDR＝0.078）等14个髓系疾病相关变异基因MF更低，PIGA在CAA组中的MF为0，其余基因的MF差异无统计学意义。

## 讨论

AA为免疫介导的骨髓衰竭性疾病，其病理机制涉及免疫系统对造血干/祖细胞的异常攻击，并伴随多种免疫相关分子及HLA基因异常[5]–[7]。造血干/祖细胞受损后，部分患者可能发展为髓系恶性疾病[8]。本研究通过比较CAA与non-AA肿瘤患者及PAA患者的体细胞变异特征，探讨CAA的分子病理基础及其潜在的髓系演变倾向。

KEGG富集分析提示CAA变异基因在JAK-STAT信号通路、钙离子信号通路及细胞外基质-受体相互作用等通路中显著富集。这些通路分别与免疫调节、细胞应激反应及骨髓微环境功能相关，提示CAA的发病机制可能兼具免疫异常、细胞应激与微环境失调等多重特征。

HLA在免疫应答中居于核心地位，其变异与免疫病理密切相关[9]，并可能影响AA患者对免疫抑制治疗的反应与预后[10]。其中，HLA-DRB1是多态性极高的Ⅱ类基因，负责将抗原呈递给CD4^+^ T细胞，其异常可影响抗原识别并触发自身免疫[11]。既往研究提示HLA-DRB1*15:01是中国北方汉族人群中AA的易感等位基因[12]。本研究发现，CAA患者中HLA-DRB1的MF显著高于non-AA肿瘤患者（FDR＝0.029），与PAA患者无显著差异，提示CAA的免疫病理中可能存在HLA-DRB1相关的异常抗原呈递，与PAA的免疫学特征具有相似性。

相对地，Ⅰ类基因HLA-A、HLA-C负责呈递内源性抗原，其异常与CD8^+^ T细胞介导的免疫攻击相关。既往研究显示，30％以上的AA患者携带HLA-Ⅰ类基因致病变异，HLA-Ⅰ类分子异常对免疫攻击的激活是PAA发病的重要环节。而本研究观察到CAA患者的HLA-A（FDR＝0.082）、HLA-C（FDR＝0.058）MF低于PAA患者，提示CAA患者承受的免疫攻击与异常激活程度整体上低于PAA患者。这与临床观察一致：CAA在化疗后发生，血细胞减少持续时间较短，对TPO-RA反应较快[2]–[3],[13]。本研究CAA患者的6个月ORR达83.3％，明显高于既往AA治疗文献报道的缓解率[14]。

与non-AA肿瘤患者相比，CAA患者经历化疗后造血系统所受的打击更严重，选择压力更严重，存活的少数异常克隆可能携带更高负荷的髓系相关变异[15]。本研究发现，CAA患者中ASXL1（FDR＝0.047）等基因的MF高于non-AA肿瘤患者，这些基因与髓系疾病进展密切相关[16]，其变异可能提示CAA具有更高的髓系疾病演变倾向。另一方面，在non-AA肿瘤患者中TP53（FDR＝0.045）和PIK3CA（FDR＝0.049）基因的MF更高。这一差异可能是因为CAA的采样时间为化疗后，而化疗可能导致这些基因MF的减低；如已有研究表明，在接受化疗的乳腺癌患者中，TP53和PIK3CA基因的MF会出现下降[17]。

与PAA患者相比，CAA患者中BRCA2（FDR＝0.068）、RUNX1（FDR＝0.080）、ATRX（FDR＝0.072）等基因的MF更高。这些基因与髓系肿瘤发生与演变相关。BRCA2变异常见于乳腺癌、卵巢癌等实体瘤[18]–[19]；RUNX1变异在卵巢癌、结直肠癌中常见，与癌细胞增殖、迁移和不良预后相关[20]；ATRX编码染色质重塑蛋白，维持端粒完整性，其变异可能促进肿瘤发生[21]。鉴于检测样本为外周血，无法区分这些突变是实体瘤克隆来源还是新生造血克隆来源，因此突变谱可能受到患者原有实体瘤背景、化疗选择性压力以及潜在造血克隆演变等多重因素的影响。

同时，CAA患者中ASXL1（FDR＝0.045）、DNMT3A（FDR＝0.078）等基因的MF低于PAA患者，PIGA等基因的MF几乎为0。一方面，ASXL1变异会导致表观遗传调控紊乱，影响造血分化，是骨髓增生异常肿瘤（MDS）和急性髓系白血病（AML）中公认的不良预后标志[16]；DNMT3A是AML、MDS的常见变异之一[22]。这些基因涉及表观遗传调控、转录调控等功能，是与髓系疾病演变相关的重要分子，其MF更低提示CAA髓系肿瘤克隆演变的程度低于PAA。另一方面，PIGA变异可作为PAA患者免疫机制的一个重要分子标志[23]，往往提示骨髓衰竭。此变异在AA患者中常见而在本组CAA患者中未见，可能归因于本组患者的病程较短。PAA发病通常有较长的潜伏期，利于变异克隆的扩增[24]；而本组所有CAA患者在化疗前血细胞计数正常，且从确诊AA至取样分析的时间较短，可能尚未积累出可检出的PIGA变异克隆，这提示ASXL1等基因较低的MF也可能与病程较短有关。相比于PAA，CAA的髓系肿瘤演变更复杂，受到肿瘤本身特性和髓系相关基因变异的共同作用。

此外，本研究中non-AA肿瘤患者及PAA患者数据来源于公开数据库，其测序平台、深度及变异筛选流程与本研究队列并不完全一致，可能对突变检出率及MF估计造成影响。因此，组间比较结果应主要视为探索性分析，用于提示潜在差异，而非严格的定量比较。

综上所述，CAA患者患者的体细胞变异谱与non-AA肿瘤患者及PAA患者存在显著差异：HLA-DRB1变异较non-AA肿瘤患者更多，HLA-A/C变异较PAA患者更少，即CAA患者的免疫异常高于non-AA肿瘤患者，低于PAA患者，但总体程度较轻；CAA患者髓系肿瘤相关基因的MF较non-AA肿瘤患者更高，提示CAA患者相比于non-AA肿瘤患者有更高的髓系演变倾向；与PAA患者相比，CAA患者髓系肿瘤转化相关基因MF的变化并不一致（部分增高，部分降低），这与CAA患者自身肿瘤史、较短病程有关，其髓系疾病演变倾向受多重因素影响。上述结果可能为理解CAA的发病机制及长期管理提供参考。

## Supplementary Material


