# Supplementary figures and images for: Somatic and immune profiling of chemotherapy-associated aplastic anemia: a comparison with primary aplastic anemia and cancer without aplastic anemia
Source: Zhonghua Xue Ye Xue Za Zhi. 2026 May;47(5):485–9. [Article in Chinese] doi: 10.3760/cma.j.cn121090-20251119-00539 (PMC13416536; doi:10.3760/cma.j.cn121090-20251119-00539)

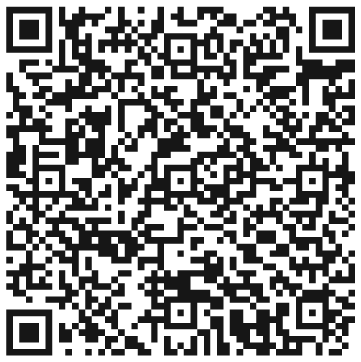

Supplement: Supplementary file 1 [file cjh-47-05-485-g003.tif]
